# Maximizers’ Susceptibility to the Effect of Frequency vs. Percentage Format in Risk Representation

**DOI:** 10.3390/bs12120496

**Published:** 2022-12-05

**Authors:** Raffaella Misuraca, Palmira Faraci, Costanza Scaffidi Abbate

**Affiliations:** 1 Department of Political Sciences and International Relations (DEMS), University of Palermo, 90134 Palermo, Italy; 2 Faculty of Human and Social Sciences, University of Enna “Kore”, 94100 Enna, Italy; 3 Department of Psychology, Educational Science and Human Movement, University of Palermo, 90133 Palermo, Italy

**Keywords:** maximizers, decision-making, frequency format, percentage format, risk

## Abstract

The present study explored the susceptibility of maximizers to the effect of the specific information format—frequency vs. percentage—in a risk assessment task. One-hundred and fourteen participants were randomized into two experimental conditions: a frequency format and a percentage format. In both conditions, participants had to rate the level of risk that a mental patient would harm someone after his discharge from a mental health facility, based on the information reported in the psychologist’s assessment for that patient. In the frequency condition, the information was presented in terms of frequencies, whereas in the percentage condition the same information was presented in terms of percentage. Our experiment showed that resolute maximizers are less affected by the specific format of the task than fearful maximizers. Thus, we conclude that resolute maximizers are more normative decision-makers. Theoretical and practical implications are discussed.

## 1. Introduction

According to the available literature on judgment and decision-making, individuals are characterized by a tendency toward either maximizing or satisficing [1,2,3,4,5].Maximizing represents the search for the best option, whereas satisficing is the search for a satisfactory, or good enough, option [6,7]. For example, in a decision about which hospital to choose for a surgery, typical maximizers, in the attempt to make the perfect choice, would engage in an exhaustive comparison of all the available hospitals to find the one that is best in all respects. Typical satisficers, instead, would evaluate only a few hospitals and then would select the first one that meets their threshold of acceptability [8].

Recent research on the maximizing/satisficing tendency has shown that maximizers are more normative decision-makers than Ref [9], see also [10]. Specifically, maximizers seem to be less susceptible to some cognitive biases, such as the framing effect, the base-rate fallacy, and the sunk-cost bias. The framing effect is the influence on the answers to a decision problem by the specific way in which the decision problem is framed or presented [11]. For example, individuals prefer to buy beef described as 75% lean compared to beef described as 25% fat [12]. Misuraca et al [9], found this effect weaker in individuals high in maximizing compared to individuals low in maximizing and attributed this finding to the greater numeracy ability of maximizers which would allow them to better process numerical information [13,14]. The base-rate fallacy consists in an error in probability judgments produced by the neglect of some crucial numerical information, and by the consideration of some unimportant descriptive information in the judgment task [15]. For example, in a task asking the probability that a randomly selected person from a group of 30 engineers and 70 lawyers is an engineer, individuals tend to judge with a higher probability that the person in an engineer rather than a lawyer if the person is described with characteristics typically associated to engineers. In other words, individuals’ answers are based on unimportant descriptive information about the person, and do not instead take into consideration the proportion of engineers and lawyers in the group from which the person is randomly drawn. Misuraca et al. [9] found this effect being stronger among individuals low in maximizing, as a consequence of maximizers’ greater ability to make judgments in accordance with the normative rules of statistical prediction. Finally, the sunk-cost bias is the tendency to pursue a suboptimal alternative merely because one has already invested money, effort, or time in it ([16], see also [17]). For example, if a person already paid for a dessert, the person tends to eat it even though they are totally full. Misuraca et al. [9] observed that individuals high in maximizing were less susceptible to the sunk-cost bias compared to individuals low in maximizing. Building on the above findings showing that individuals high in maximizing are more normative decision-makers than individuals low in maximizing, the aim of this paper is to further investigate the normative skills of maximizers. In particular, we extended the effect found by Misuraca et al. [9] to another well-established cognitive bias: the frequency vs. percentage format effect ([18,19,20,21,22,23]; see also [24]). In line with the assumption that maximizers have superior decision-making skills [9], we hypothesize that individuals high in maximizing are less susceptible to the effect of the specific format (frequency vs. percentage) of the task than individuals low in maximizing.

## 2. Experiment. Maximizing and Susceptibility to the Frequency vs. Percentage Format in Risk Judgments

Scientific evidence suggests that the specific format in which information is presented profoundly affects judgments and decision-making ([22], see also [24]). In particular, Slovic, Monahan, and MacGregor [22] showed that forensic psychologists and psychiatrists who were asked to judge the likelihood that a patient hospitalized with mental disorder would commit an act of violence within six months after discharge from the hospital, differed in their judgments depending on the specific format (percentage versus frequency) in which the information was presented: When the information was presented in terms of frequencies (e.g., 10 out of 100) the risk that the patient would have committed a crime was judged as higher compared to a scenario where the same information was presented in terms of probabilities (e.g., 10% chance). Building of these findings, the goal of our study is to investigate whether decision-makers high and low in maximizing differ in their risk representation (and thus in their judgments), as a consequence of the specific format in which information is presented. We expect that the format of presentation would affect judgments less among individuals high in maximizing than among individuals low in maximizing.

### 2.1. Method

The participants included one-hundred and fourteen (*M_age_* = 22.46; 84.2% female) undergraduate volunteers from an Italian university. The participants were randomized into two experimental conditions: frequency format (*N*_1_ = 61); and percentage format (*N*_2_ = 53). The task was an adapted version of the scenario used by Slovic et al. ([22], see also [21]). In both conditions, participants read a psychologist’s assessment concerning the evaluation for discharge of a mental patient, Mr. Mario Rossi, from the mental health facility. In the frequency condition, participants had the information presented in terms of frequencies (*Of every 100 patients similar to Mr. Rossi, 10 are estimated to commit an act of violence to others during the first several months after discharge*). In the percentage condition, instead, the same information was presented in terms of percentage (*Of every 100 patients similar to Mr. Rossi, 10% are estimated to commit an act of violence to others during the first several months after discharge*). In both conditions, participants’ task was to rate, on a scale ranging from 1 (low risk) to 7 (high risk), the level of risk that Mr. Rossi would harm someone after his discharge (see Appendix A). Higher differences between the frequency and the percentage version indicated a greater susceptibility to the specific presentation format.

Participants’ tendency towards maximization was then assessed with the 11 maximizing items of the Decision-Making Tendency Inventory ([25] see Appendix B), in which the answers ranged from 1 (=strongly disagree) to 7 (=strongly agree). This scale distinguishes between two independent facets of maximizing: the *resolute* and the *fearful* maximizing. The former seems to have a clear idea of which goals to achieve and evaluate a large number of options in order to achieve those goals, whereas the latter seem to process a large number of options not because they know what they want, but just because they fear making wrong decisions. The resolute maximizers, thus, best mirror the classical definition of maximizers in literature [6,7]. A maximizing score was calculated for each participant by summing their responses to the 11 maximizing items. The higher the score, the higher the tendency towards maximization. A resolute maximizing score and a fearful maximizing score was calculated for each participant by summing their responses to the resolute maximizing (items 1–5 in Appendix B) and fearful maximizing items (items 6–11 in Appendix B), respectively. The higher the score, the higher the tendency towards each facet. Participants then completed some demographic items regarding age and gender.

### 2.2. Results and Discussion

**Figure 1:**
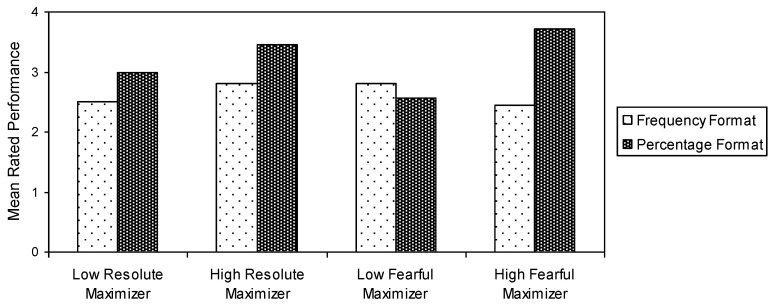
Mean risk ratings by both resolute and fearful maximizers in the frequency vs. percentage format.

The averaged maximizer score was 50.15 (median = 50; range = 30−74; DS = 9.04). The mean resolute maximizer score was 25.78 (median = 26; range = 14−35; DS = 4.60). The mean fearful maximizer score was 24.37 (median = 24; range = 12−40; DS = 6.48). Findings derived from the univariate analysis of variance (ANOVA) of the risk ratings highlighted the frequency vs. percentage format effect: mean risk ratings were 2.66 and 3.26 for the frequency and percentage format, respectively, *F* (1, 113) = 3.54, *p* < 0.05. According to the performed General Linear Model analysis, neither resolute maximizers nor fearful maximizers had a significant main effect, *F* (1, 113) = 0.7, *p* = 0.399. The interaction between resolute maximizer and format was not significant, *F* (1, 113) = 0.4, *p* = 0.554, whereas the interaction between fearful maximizer and format was significant *F* (1, 113) = 6.7, *p* = 0.01. As a result, resolute maximizers did not show susceptibility to the frequency vs. percentage format effect, whereas fearful maximizer scored higher risk ratings when a percentage representation of risk was presented rather than when a frequency representation of risk was presented (see Figure 1).

## 3. General Discussion

The present study tested the hypothesis that maximizers are less susceptible to the effect of the specific format (frequency vs. percentage) in risk judgements than individuals low in maximizing. The scale that we used to assess the maximizing tendency distinguishes between two facets of maximization: the resolute and the fearful maximizing tendency. Whereas the former have a clear idea of the goals that they want to achieve and process a considerable amount of information to achieve their goals, the latter process a considerable amount of information while deciding mostly because they fear making wrong decisions rather than because they have a clear idea of the goals that they want to achieve. In other words, the resolute maximizers best mirror the maximization concept originally proposed by Simon [6,7]. In a task asking to make judgments about a patient’s violence risk, we found that resolute maximizers were more consistent with their judgments regardless of the specific format (frequency vs. percentage) of the task compared to fearful maximizers. Fearful maximizers, instead, were more inconsistent with their judgments depending on the specific format of the task, expressing a higher perceived risk when the task was formulated in terms of probabilities rather than frequencies. The present findings align well with Misuraca et al.’s [9] work showing that maximizers (in this specific case, the resolute maximizers) have greater ability to think normatively. Our results align well, also, with recent work showing that maximizing can be a favorable decision-making style since it is related to positive outcomes, such as the ability to consider the long-term effects of their current behaviors [26].

The present findings have both theoretical and practical implications. From a theoretical point of view, they add to the growing knowledge regarding individual differences in decision-making, supporting the assumption that the resolute maximizing tendency produces optimal outcomes, since it is associated with less susceptibility to the effect of the frequency vs. percentage format of the task. Furthermore, our data pose some boundaries to the current general anti-maximizer bias in most of the available literature, that describes maximizers as individuals characterized by maladaptive decision-making styles and low levels of well-being [3,27,28,29,30,31,32,33]. In contrast with prior literature, our findings suggest that there are virtues of being a maximizer, rather than just vices. One of these virtues is less-biased decision-making.

From a practical point of view, our findings may provide suggestions and insights for the development of new decision aids in accordance with the specific decision-making tendency of the individuals who are supposed to be helped (see [34,35]). As pointed out by Misuraca et al. [9], individuals low in resolute and fearful maximizing may benefit from a decision aid different from that benefiting individuals high in these maximizing tendencies (see also [25,36]). Another important practical implication of our findings concerns the hiring process: since resolute maximizers make more optimal decisions, it might be wiser for organizations to select more resolute maximizers for tasks that require them to make strategical decisions. It is also interesting to explore whether a team’s performance can be improved by the inclusion of both resolute and fearful maximizers in order to leverage employees’ cognitive strengths.

In sum, our study supports the conclusion that resolute maximizers, which correspond to the original definition of maximizers [6,7], are generally capable of making rational decisions. Further research should be aimed at exploring the generality of this conclusion across different cultures and domains, such as medical, financial, educational, managerial, and so on. Additionally, a search for further virtues of the maximizing tendency might represent a promising direction for future research.

## Data Availability

https://docs.google.com/spreadsheets/d/1-2D9SMuQ5PVGcS8DJN-tPG9ZcL6l-ivb/edit?usp=sharing&ouid=108225773687535081449&rtpof=true&sd=true, (accessed on 30 November 2021).

## Appendix A. Risk Assessment Task (Adapted from Slovic et al., 2000)

A patient—Mr. Mario Rossi—has been evaluated for discharge from an acute civil mental health facility where he has been treated for the past several weeks. A psychologist whose professional opinion you respect has done a state-of-the-art assessment of Mr. Rossi. Among the conclusions reached in the psychologist’s assessment is the following:

### Appendix A.1. Frequency Format

Of every 100 patients similar to Mr. Rossi, 10 are estimated to commit an act of violence to others during the first several months after discharge.

Please rate, on a scale ranging from 1 (low risk) to 7 (high risk), the level of risk that Mr. Rossi would harm someone.

### Appendix A.2. Percentage Format

Of every 100 patients similar to Mr. Rossi, 10% are estimated to commit an act of violence to others during the first several months after discharge.

Please rate, on a scale ranging from 1 (low risk) to 7 (high risk), the level of risk that Mr. Rossi would harm someone.

## Appendix B. The 11 Maximization Items in the Decision Making Tendency Inventory (Misuraca et al., 2015)

Resolute Maximizing Subscale
1. In studying or working, I always set the highest targets.
2. No matter what I do, I have the highest standards for myself.
3. I never settle for second best.
4. I never settle.
5. No matter how satisfied I am with my job, it’s only right for me to be on the lookout for better opportunities.
Fearful Maximizing Subscale
6. In all decisions that affect my work or studying, I am always afraid of not choosing the best options.
7. Renting videos is really difficult. I am always struggling to pick the best one.
8. When shopping, I have a hard time finding clothing that I really love.
9. Whenever I am faced with a choice, I try to imagine what all the other possibilities are, even ones that are not present at the moment.
10. When I am in the car listening to the radio, I often check other stations to see if something better is playing, even if I am relatively satisfied with what I am listening to.
11. To assure that I get the best deal, I always consult consumer product reviews before buying.
